# Proteasome Composition and Activity Changes in Cultured Fibroblasts Derived From Mucopolysaccharidoses Patients and Their Modulation by Genistein

**DOI:** 10.3389/fcell.2020.540726

**Published:** 2020-10-20

**Authors:** Karolina Pierzynowska, Lidia Gaffke, Elżbieta Jankowska, Estera Rintz, Julia Witkowska, Ewa Wieczerzak, Magdalena Podlacha, Grzegorz Węgrzyn

**Affiliations:** ^1^Department of Molecular Biology, Faculty of Biology, University of Gdañsk, Gdañsk, Poland; ^2^Department of Biomedical Chemistry, Faculty of Chemistry, University of Gdañsk, Gdañsk, Poland

**Keywords:** mucopolysaccharidosis, proteasome, genistein, transcriptomics, ubiquitinated proteins

## Abstract

In this study, we have asked whether proteasome composition and function are affected in cells derived from patients suffering from all types of mucopolysaccharidosis (MPS), an inherited metabolic disease caused by accumulation of undegraded glycosaminoglycans (GAGs). Moreover, we have tested if genistein, a small molecule proposed previously as a potential therapeutic agent in MPS, can modulate proteasomes, which might shed a new light on the molecular mechanisms of action of this isoflavone as a potential drug for macromolecule storage diseases. Significant changes in expression of various proteasome-linked genes have been detected during transcriptomic (RNA-seq) analyses in vast majority of MPS types. These results were corroborated by demonstration of increased proteasomal activities in MPS cells. However, GAGs were not able to stimulate the 26S proteasome *in vitro*, suggesting that the observed activation in cells is indirect rather than arising from direct GAG-proteasome interactions. Genistein significantly reduced proteasomal activities in fibroblasts derived from patients suffering from all MPS types, while its effects on *in vitro* 26S proteasome activity were negligible. Unexpectedly, levels of many proteasomal subunits were increased in genistein-treated MPS cells. On the other hand, this ostensible discrepancy between results of experiments designed for estimation of effects of genistein on proteasome activities and abundance of proteasomal subunits can be explained by demonstration that in the presence of this isoflavone, levels of ubiquitinated proteins were decreased. The genistein-mediated reduction of proteasomal activities might have beneficial effects in cells of MPS patients due to potential increasing of residual activities of defective lysosomal enzymes which would otherwise be subjected to efficient ubiquitination and proteasomal degradation as misfolded proteins. These results indicate another activity of genistein (apart from previously demonstrated reduction of GAG synthesis efficiency, stimulation of lysosomal biogenesis, and activation of the autophagy process) which can be beneficial in the use of this small molecule in treatment of MPS.

## Introduction

Mucopolysaccharidoses (MPS) consist of a group of macromolecule storage disorders in which mutations in single genes coding for enzymes responsible for degradation of glycosaminoglycans (GAGs) cause accumulation of these compounds in lysosomes (Tomatsu et al., 2018). Depending on specific enzymatic deficits and storage of particular GAGs [dermatan sulfate (DS), heparan sulfate (HS), keratan sulfate (KS), chondroitin sulfate (CS), hyaluronate), there are 11 types and subtypes of MPS distinguished (Tomatsu et al., 2018). They are characterized briefly in Table 1. MPS are severe diseases, with multiple symptoms and progressive course, with average expected life span of about two decades. Currently, hematopoietic stem cell transplantation therapy (HSCT) and enzyme replacement therapy (ERT) are available for some MPS types (Chen et al., 2019; Taylor et al., 2019), but only some of many medical problems can be solved by using these therapeutic methods. Gene therapy is being developed for some MPS types, but this option still needs confirmation of efficacy (Fraldi et al., 2018; Sawamoto et al., 2018). Other potential therapies for MPS have also been tested, with small molecule-mediated substrate reduction therapy (SRT) giving promising results in experiments on animal models (Malinowska et al., 2010; Jakóbkiewicz-Banecka et al., 2011; Derrick-Roberts et al., 2017; Gaffke et al., 2018).

**TABLE 1:** MPS types and subtypes (according to Tomatsu et al., 2018), and previously reported proteasomal changes in MPS cells (references provided in the last column).

MPS type	Mutated gene	Deficient enzyme	Primary stored GAG(s)^*a*^	Reported major proteasomal changes in MPS	References
I	*IDUA*	α-L-iduronidase	HS, DS	Enhanced expression of genes coding for proteasomal proteins	Khalid et al., 2016
II	*IDS*	iduronate-2-sulfatase	HS, DS	Rapid proteasomal (ERAD) degradation of mutant (A85T) iduronate-2-sulfatase, and enhanced residual activity of this enzyme after ERAD inhibition	Osaki et al., 2018
				Rapid proteasomal degradation of the mutant form (W337X) of iduronate-2-sulfatase	Marazza et al., 2020
IIIA	*SGSH*	N-sulfoglucosamine sulfhydrolase	HS	Rapid proteasomal degradation of mutant (S298P) N-sulfoglucosamine sulfhydrolase	Muschol et al., 2011
				Enhanced proteasomal degradation of cysteine string protein α (CSPα), and normalization of CSPα levels in neurons after proteasome inhibition	Sambri et al., 2017
				Elevated levels of the 19S proteasomal subunit	Beard et al., 2017
IIIB	*NAGLU*	α-N-acetylglucosaminidase	HS	Rapid proteasomal degradation of synaptophysin and re-establishing of normal synaptophysin levels after inhibition of proteasomal functions	Vitry et al., 2009
IIIC	*HGSNAT*	Acetyl-CoA: α-glucosaminide acetyltransferase	HS	None	N/A
IIID	*GNS*	N-acetylglucosamine-6-sulfatase	HS	None	N/A
IVA	*GALNS*	N-acetylgalactosamine-6-sulfatase	KS, CS	None	N/A
IVB	*GLB1*	β-galactosidase-1	KS	None	N/A
VI	*ARSB*	N-acetylgalactosamine 4-sulfatase	DS, CS	None	N/A
VII	*GUSB*	β-glucuronidase	HS, DS, CS	None	N/A
IX	*HYAL1*	Hyaluronidase-1	Hyaluronic acid	None	N/A

*^*a*^Secondary DS storage is observed in all subtypes of MPS III. CS, chondroitin sulfate; DS, dermatan sulfate; HS, heparan sulfate; KS, keratan sulfate; N/A, not applicable.*

Among small molecules tested in SRT, rhodamine B {[9-(2-carboxyphenyl)-6-diethylamino-3-xanthenylidene]-diethylammonium chloride} and genistein [5,7-dihydroxy-3-(4-hydroxyphenyl)chromen-4-one] appear to be the most promising ones (Gaffke et al., 2018). Interestingly, the latter compound, apart from lowering efficiency of GAG synthesis, thus, facilitating achievement of a balance between production and degradation of these compounds, has been found to stimulate lysosomal biogenesis by activation of the transcription factor EB (Moskot et al., 2014). This suggested that genistein may improve metabolism in MPS cell through modulating various processes. In this light, it is worth noting that recent studies indicated global changes in various processes occurring in cells derived from MPS patients (Gaffke et al., 2019, 2020; Brokowska et al., 2020; Pierzynowska et al., 2020b).

Among the cellular processes affected in MPS, there is autophagy, one of major pathways of degradation of macromolecules in eukaryotic cells (Pierzynowska et al., 2020a). In fact, genistein has been reported to stimulate autophagy, and it was suggested that pharmacological activation of the autophagy process can be considered as a therapeutic approach in various storage diseases, including those with neurodegenerative components (Pierzynowska et al., 2018a,b, 2019). On the other hand, the second major process of macromolecule degradation in cells is proteasome-mediated proteolysis. The ubiquitin-proteasome system is a complex (consisting in over a thousand of components) cellular machinery responsible for degradation of superfluous cytoplasmic proteins (Kudriaeva and Belogurov, 2019). Because of its global effects on cellular metabolism, modulation of proteasome activity has been considered as a therapeutic approach in a large number of various diseases (Thibaudeau and Smith, 2019). Since genistein has been reported as a compound that might influence activity of the proteasome (Kazi et al., 2003; Shim, 2011), in this work we have asked whether proteasome composition and function are affected in cells derived from patients suffering from various MPS types, and if genistein can modulate them, which might shed a new light on the molecular mechanisms of action of this small molecule as a potential drug for macromolecule storage diseases. To date, only relatively few reports addressed the problem of proteasomal activities in MPS cells, and each particular article concerned only a single MPS type. Major findings described in these reports are summarized in Table 1. We have employed both tanscriptomic and biochemical methods to test whether there are significant changes in composition of the proteasome and its functions in fibroblasts of all MPS types. Although previous transcriptomic analyses indicated that expressions of genes coding for proteins involved in various functions/processes can be changed in MPS cells, including apoptosis (Brokowska et al., 2020), cell activation (Rintz et al., 2020), cell metabolic processes (Gaffke et al., 2020), and even processes leading to behavioral disorders (Pierzynowska et al., 2020b), proteasome composition and functions were not assessed in such an experimental system.

## Materials and Methods

### Cell Lines

In this study, fibroblasts commercially purchased from the NIGMS Human Genetic Cell Repository at the Coriell Institute for Medical Research were used (this Institute contains all documents related to bioethical issues). Following cell lines were employed: HDFa – control fibroblasts derived from a healthy person; the second control fibroblast cell line (CTRL-1) from a healthy person (age: 51 years, sex: male, race: Caucasian), used in reverse transcription – quantitative real-time polymerase chain reaction (RT-qPCR) experiments (Pierzynowska et al., 2020b); MPS I – fibroblasts (NIGMS Cat. No. GM00798) from a patient (age: 1 year, sex: female, race: Caucasian) bearing mutations c.G1293A/G1293A (p.Trp402Ter/p.Trp402Ter) in the *IDUA* gene; MPS II – fibroblasts (NIGMS Cat. No. GM13203) from a patient (age: 3 years, sex: male, race: Caucasian) bearing a mutation c.208insC (p.His70ProfsTer29) in the *IDS* gene (hemizygote); MPS IIIA – fibroblasts (NIGMS Cat. No. GM00879) from a patient (age: 3 years, sex: female, race: Caucasian) bearing mutations c.G1351A/G746A (p.Glu447Lys/p.Arg245His) in the *SGSH* gene; MPS IIIB – fibroblasts (NIGMS Cat. No. GM00156) from a patient (age: 7 years, sex: male, race: Caucasian) bearing mutations c.C1876T/C1876T (p.Arg626Ter/p.Arg626Ter) in the *NAGLU* gene; MPS IIIC – fibroblasts (NIGMS Cat. No. GM05157) from a patient (age: 8 years, sex: male, race: unknown) bearing unidentified mutations in the *HGSNAT* (diagnosed on the basis of estimation of urinary GAG levels and activity of the corresponding enzyme in plasma); MPS IIID – fibroblasts (NIGMS Cat. No. GM05093) from a patient (age: 7 years, sex: male, race: Asian-Indian) bearing mutations c.C1063T/C1063T (p.Arg355Ter/p.Arg355Ter) in the *GNS* gene; MPS IVA – fibroblasts (NIGMS Cat. No. GM00593) from a patient (age: 7 years, sex: female, race: Caucasian) bearing unidentified mutations in the *GALNS* gene (diagnosed on the basis of estimation of urinary GAG levels and activity of the corresponding enzyme in plasma); MPS IVB – fibroblasts (NIGMS Cat. No. GM03251) from a patient (age: 4 years, sex: female, race: Caucasian) bearing mutations c.TG851-852CT/G1561T (p.Trp273Leu/p.Trp509Cys) in the *GLB1* gene; MPS VI – fibroblasts (NIGMS Cat. No. GM03722) from a patient (age: 3 years, sex: female, race: Black) bearing unidentified mutations in the *ARSB* gene (diagnosed on the basis of estimation of urinary GAG levels and activity of the corresponding enzyme in plasma); MPS VII – fibroblasts (NIGMS Cat. No. GM00121) from a patient (age: 3 years, sex: male, race: African American) bearing mutations c.G1881T/G1068A (p.Trp627Cys/p.Arg356Ter) in the *GUSB* gene; MPS IX – fibroblasts (NIGMS Cat. No. GM17494) from a patient (age: 14 years, sex: female, race: unknown) bearing unidentified mutations in the *HYAL1* gene (diagnosed on the basis of estimation of urinary GAG levels and activity of the corresponding enzyme in plasma). Each fibroblast line has been used to establish independent cultures, each at the passage between 4th and 15th.

### Isolation and Purification of RNA

Four biological repeats of each RNA isolation and purification procedure were performed by using four independent cultures of every cell line, each from different passage. In each experiment, 5 × 10^5^ fibroblasts were seeded on 10 cm-diameter plate, and cultured in the DMEM medium supplemented with antibiotics and 10% fetal bovine serum (FBS), at 37°C, 95% humidity, and saturation with 5% CO_2_. Guanidine isothiocyanate, beta-mercaptoethanol, and the QIAshredder column were used for cell lysis. RNA was extracted by using the RNeasy Mini kit (Qiagen) and Turbo DNase (Life Technologies), and employing the procedures described in manuals provided by manufacturers. Using the Agilent 2100 Bioanalyzer System with RNA Nano Chips (Agilent Technologies), quality of RNA samples was assessed.

### RNA-seq Analysis

The RNA-seq analyses were performed exactly as described previously (Gaffke et al., 2020; Pierzynowska et al., 2020b). Briefly, Illumina TruSeq Stranded mRNA Library Prep Kit was used to prepare the mRNA libraries. Then, the cDNA libraries were sequenced employing a HiSeq4000 (Illumina, San Diego, CA, United States). Following parameters were used: PE150 (150 bp paired-end) and minimum 40 × 10^6^ of raw reads. This gave a minimum of 12 Gb of raw data per each sample. FastQC version v0.11.7 was used for quality assessment. Raw readings were mapped to the GRCh38 human reference genome from the Ensembl database. Hisat2 v. 2.1.0 program was used for this procedure. Cuffquant and Cuffmerge programs in version 2.2.1 and the GTF Homo_sapiens.GRCh38.94.gtf file from the Ensembl database were used to calculate the expression levels. The Cuffmerge program was started with the library-norm-method classic-fpkm parameter normalizing the expression values by means of the FPKM algorithm. Transcript annotation and classification was performed using the BioMart interface for the Ensembl gene database. RNA-seq data are deposited at NCBI Sequence Read Archive (SRA), under the accession no. PRJNA562649.

### Determination of Proteasomal Activities in Cells

To determine chymotrypsin-like, trypsin-like, and caspase-like proteasomal activities in tested cell lines, the luminescent Cell-Based Proteasome-Glo^TM^ Assays (Promega, Madison, WI, United States), were used according to manufacturer’s instruction. Briefly, 1 × 10^4^ cells were seeded in each well of 96-well plate (before inoculation, the cells were washed three times to remove any trypsin contamination from a previous passage), and cultured in the DMEM medium supplemented with antibiotics and 10% FBS, at 37°C, 95% humidity, and saturation with 5% CO_2_ for 24 h. Then, the cultures were treated with either PBS (control; volume equal to that used for DMSO, genistein and MG-132), DMSO (final concentration 0.05%), genistein (50 μM) or MG-132 (10 μM), and following another 24 h incubation, substrates were added and luminescence was measured as described in the manufacturer’s instruction.

### Measurement of Proteasomal Activities *in vitro*

Purified human 26S proteasome (h26S) was purchased from Enzo Life Sciences Inc. (New York, NY, United States). The enzyme activity was assayed as described by Kisselev and Goldberg (2005). Briefly, the stock proteasome (1 mg/ml) was thawed on ice immediately before measurements and diluted to 8 μg/ml in the assay buffer (50 mM Tris/HCl pH 7.6, 40 mM KCl, 5 mM MgCl_2_, 1 mM DTT) containing 2 mM ATP. Suc-LLVY-AMC substrate (Enzo Life Sciences, New York, NY, United States) was used to probe the h26S chymotrypsin-like activity. A total of 50 mM stock solution of this model fluorogenic peptide was prepared in DMSO and diluted to 200 μM with the assay buffer containing 0.1 mg/ml bovine serum albumin (BSA). Genistein and GAGs were dissolved in DMSO and 4 × concentrated solution of each work concentration was prepared by diluting with the assay buffer. The enzymatic reaction was carried out in 96-well plates. Each well contained 25 μl of the h26S solution, 50 μl of the substrate solution and 25 μl of either genistein or GAG solution. The total concentration of DMSO in the final reaction mixtures never exceeded 3% (vol/vol). The final substrate concentration was 100 μM, the proteasome content 0.2 μg in 50 mM Tris/HCl pH 7.6, 40 mM KCl, 5 mM MgCl_2_, 1 mM DTT, 0.5 mM ATP, 50 μg/ml BSA (BSA was added to minimize the enzyme adsorption to the well surface). As a negative control MG-132 was used at the final concentration of 100 μM. Measurements were carried out using Tecan Infinite 200Pro plate reader (Tecan Group Ltd., Mannedorf, Zurich, Switzerland). The release of an AMC reporter group was followed by measuring fluorescence emission in 2 min intervals for up to 60 min at 37°C. The peptidolytic activity was calculated as nanomoles of the released AMC product per mg of proteasome per second. All data are presented as mean ± SD from three independent experiments.

### Western-Blotting

Fibroblasts (6 × 10^5^) were seeded on plates (10 cm in diameter) and allowed to attach overnight in the DMEM medium supplemented with antibiotics and 10% FBS, at 37°C, 95% humidity, and saturation with 5% CO_2_. Cells were treated with either 0.05% DMSO (control), 50 μM genistein or 10 mM MG-132 for 24 h. For cell lysis, the following solution was used: 1% Triton X-100, 0.5 mM EDTA, 150 mM NaCl, 50 mM Tris, pH 7.5, and a mixture of protease and phosphatase inhibitors (Roche Applied Science, Penzberg, Germany. The mixture was cleared by centrifugation in a microfuge. For protein separation and detection of specific proteins, the WES system (WES – Automated Western Blots with Simple Western; ProteinSimple, San Jose, CA, United States), with 12–230 kDa Separation Module and Anti-Mouse Detection Module, was used according to the manufacturer’s instruction. Mouse monoclonal antibodies against specific proteasomal subunits were purchased from Enzo Biochem Inc. (New York, NY, United States) (BML-PW8900-0001 and BML-PW8900-0001 for kits containing antibodies against α and β subunits, respectively). Anti-ubiquitin antibodies (Ubiquitin (E4I2J) Rabbit mAb; Cell Signaling Technology, Leiden, Netherlands) were used to detected ubiquitinated proteins in western-blotting. Staining with monoclonal anti-GAPDH-peroxidase antibody (Merck, Darmstadt, Germany) was used as an internal control to normalize the amounts of proteins.

### Assessment of Changes in Levels of Specific Ubiquitinated Proteins

Changes in levels of ubiquitinated forms of 49 proteins have been assessed using the Proteome Profiler Human Ubiquitin Array (R&D Systems Inc., Minneapolis, MN, United States), according to the manufacturer’s instruction. Cell lysates were prepared from fibroblasts cultured as described in section “Western-Blotting.”

### Reverse Transcription – Quantitative Real-Time Polymerase Chain Reaction

Total RNA (isolated and purified from fibroblasts as described in section “Isolation and Purification of RNA”) was used for reverse transcription with iScript Reverse Transcription Supermix for RT-qPCR (Bio-Rad, Hercules, CA, United States), using procedures described in the manufacturer’s instructions. RT-qPCR was performed with specific primers, listed in Supplementary Table 1. The CFX96 Touch Real-Time PCR Detection System (Bio-Rad, Hercules, CA, United States) was employed. Levels of assessed mRNAs were calculated by the 2^–ΔΔ*C(T)*^ method.

### Statistical Analyses

In transcriptomic studies, statistical significance was analyzed using one-way analysis of variance (ANOVA) on log_2_(1 + x) values which have normal continuous distribution. The false discovery rate (FDR) was estimated using the Benjamini–Hochberg method. For comparisons between two groups, *post hoc* Student’s *t*-test with Bonferroni correction was employed. R software v3.4.3 was employed to conduct all statistical analyses. Statistical significance was assessed at FDR < 0.1 and *p* < 0.1 which are standard parameters for transcriptomic analyses with at least four biological repeats (Brokowska et al., 2020; Gaffke et al., 2020; Pierzynowska et al., 2020b).

In other experimental studies, the normality of the distribution of variables was checked with the Kolmogorov–Smirnov test, and the homogeneity of the variances with the Levene test. For some parameters, the outcome of the Kolmogorov–Smirnov test indicated that the data was not distributed normally; in these cases, we used non-parametric tests for further analysis. Chymotrypsin-like, trypsin-like, and caspase-like 26S proteasomal activities as well as effects of GAGs and genistein on activity of h26S *in vitro* were evaluated using Kruskal–Wallis and Dunn *post hoc* test. For other analyses, one-way ANOVA and Tukey’s *pos hoc* test were performed. Multiple comparisons between MPS fibroblasts and control cells were performed using the Dunnett’s test.

## Results

### Transcriptomic Analyses

To test if proteasome composition and activity can be changed in MPS, we have started our study from performing large-scale transcriptomic analysis. To make the analysis as broad as possible, considering that there are 11 known types and subtypes of MPS, we have used fibroblast lines derived from patients suffering from all these types/subtypes (i.e., MPS I, II, IIIA, IIIB, IIIC, IIID, IVA, IVB, VI, VII, and IX). As controls, human dermal fibroblasts (the HDFa cell line) were employed. This kind of analysis has been demonstrated previously to be effective in considering changes occurring in various cellular processes in MPS (Gaffke et al., 2020; Pierzynowska et al., 2020b). Following *in vitro* cultivation of investigated cell lines, total mRNA was isolated, and transcriptomic analysis has been performed by RNA sequencing (RNA-seq). In these experiments, four biological repeats were conducted for each cell line (i.e., four independent cultures were performed, and each culture derived from different passage). For bioinformatic analyses, we have used RNA-seq data deposited in NCBI Sequence Read Archive, SRA (accession number: PRJNA562649). The qualities of the isolated RNAs and sequencing procedures were verified by considering numbers of reads. They were between ∼40 and ∼60 million (see Gaffke et al., 2020 for details).

**FIGURE 1:**
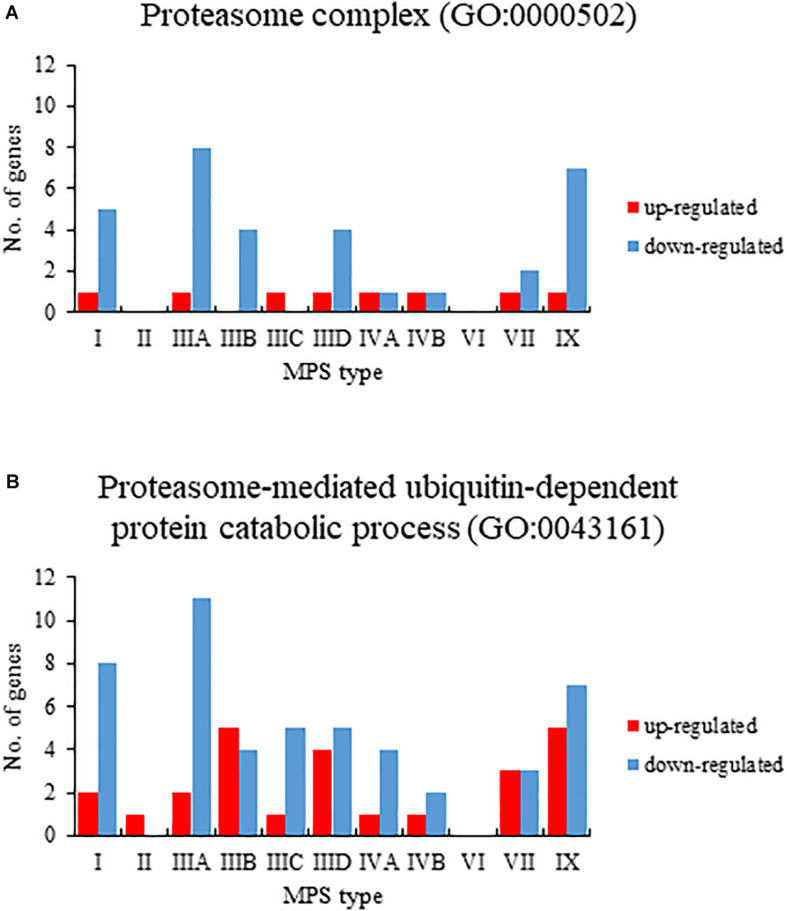
Number of up (red)- and down (blue)-regulated transcripts (at FDR < 0.1; *p* < 0.1), grouped in GO terms proteasome complex (GO:0000502) **(A)** and proteasome-mediated ubiquitin-dependent protein catabolic process (GO:0043161) **(B)**, in different types of MPS relative to control cells (HDFa), as revealed by transcriptomic (RNA-seq) analyses.

When assessing changes in levels of transcripts in MPS cells relative to control cells, we have performed Gene Ontology (GO) analysis (using the QuickGO database). When considering GO terms related to proteasome composition and function, we have found that expression of considerable number of corresponding genes was significantly changed in many MPS types/subtypes relative to control cells. Both up- and down-regulated genes belonged to GO terms: proteasome complex (GO:0000502) and proteasome-mediated ubiquitin-dependent protein catabolic process (GO:0043161) (Figure 1). The highest number of miss-regulated proteasome-related genes was detected in MPS IIIA (9 and 13 in GO:0000502 and GO:0043161, respectively), MPS IX (8 and 12), and MPS I (6 and 10). There were only a few MPS types in which no significant changes in levels of proteasome-related transcripts could be detected, namely MPS II and MPS IIIC for GO:0000502, and MPS VI for both GO:0000502 and GO:0043161 (Figure 1). These results indicated that both composition and function of proteasome can be affected in cells derived from patients suffering from most MPS types.

**FIGURE 2:**
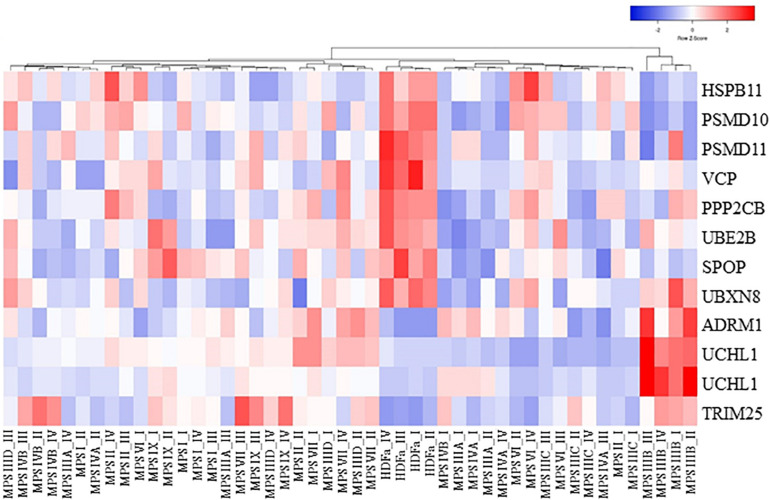
Clustered heat map of proteasome-related transcripts indicating those which levels are significantly changed in at least three MPS types/subtypes relative to the control cells. The color codes and scale are indicated.

We asked what genes coding for proteins involved in proteasome composition/function revealed changed expression in several MPS types. Hence, we have chosen those which transcripts were up- or down-regulated in at least three different MPS types/subtypes. This criterium was fulfilled by following 11 genes: *HSPB11, PSMD10, PSMD11, VCP, PPP2CB, UBE2B, SPOP, UBXN8, ADRM1, UCHL1* (two alternative transcripts), and *TRIM25*. The transcriptomic analysis for them is presented as a heat-map (Figure 2), and quantitative changes are demonstrated in Table 2 (with detailed statistical analysis shown in Supplementary Table 2). These results indicated that some changes in expression of proteasome-related genes may be common for at least several MPS types, and the most pronounced changes are in MPS IIIB.

**TABLE 2:** Proteasome-related transcripts which levels were significantly changed [false discovery rate (FDR) < 0.1, *p* < 0.1] in at least three MPS types/subtypes relative to the control cells.

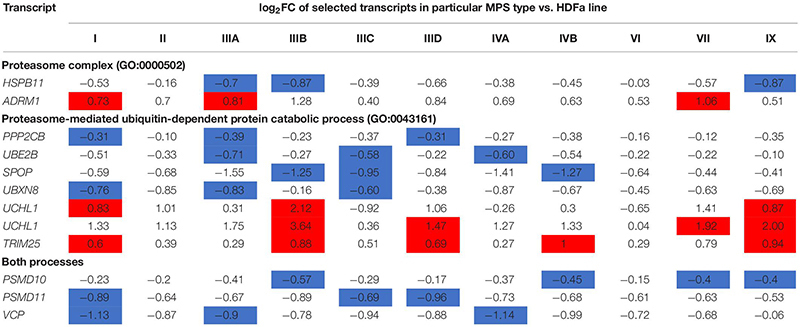

*Up-regulated transcripts are marked in red, and down-regulated transcripts are marked in blue. Values not marked by color indicate results for which no statistically significant differences were detected between MPS and control (HDFa) cells.*

In the next step, we have assessed genes which expression is particularly strongly changed in MPS cells relative to the control. When assuming the threshold of twofold change (i.e., log_2_ fold change (FC) > 1), we have identified following 11 genes fulfilling this criterium in at least one MPS type: *UCHL1, FBXO38, TRIM25, CUL4A, VCP, SPOP, PSMD2, PSMD13, PSMB8, PSMB9, PSME2, HSPB1*, and *ADRM1* (Table 3; with detailed statistical analysis shown in Supplementary Table 3). Volcano plots indicating the most significant changes are shown in Supplementary Figures 1, 2. Thus, we assumed that such significant changes might indicate considerable disturbances in the functioning of the proteasomal system in at least some MPS types.

**TABLE 3:** Proteasome-related genes which transcripts occurring at levels with log_2_FC > 1.0 in particular types of MPS relative to control cells (HDFa).

**Transcript**	**The most changed transcripts (log_2_FC > 1) in particular MPS type vs. HDFa line**										
	
	**I**	**II**	**IIIA**	**IIIB**	**IIIC**	**IIID**	**IVA**	**IVB**	**VI**	**VII**	**IX**
**Proteasome complex (GO:0000502)**											
*PSMD13*	▼		▼								
*PSME2*				▼							
*HSPB1*						▲		▲			
**Proteasome-mediated ubiquitin-dependent protein catabolic process (GO:0043161)**											
*SPOP*				▼				▼			
*RPS27A*							▼				
*UCHL1*				▲							
*UCHL1*				▲		▲				▲	▲
*UCHL1*				▲							
*FBXO38*						▲					
*TRIM25*								▲			
*CUL4A*					▲						
**Both processes**											
*VCP*	▼						▼				
*PSMD2*	▼		▼								
*PSMB8*							▲				
*PSMB9*				▼							
*ADRM1*										▲	

*Up-arrowed and down-arrowed symbols indicate up- and down-regulation, respectively.*

**FIGURE 3:**
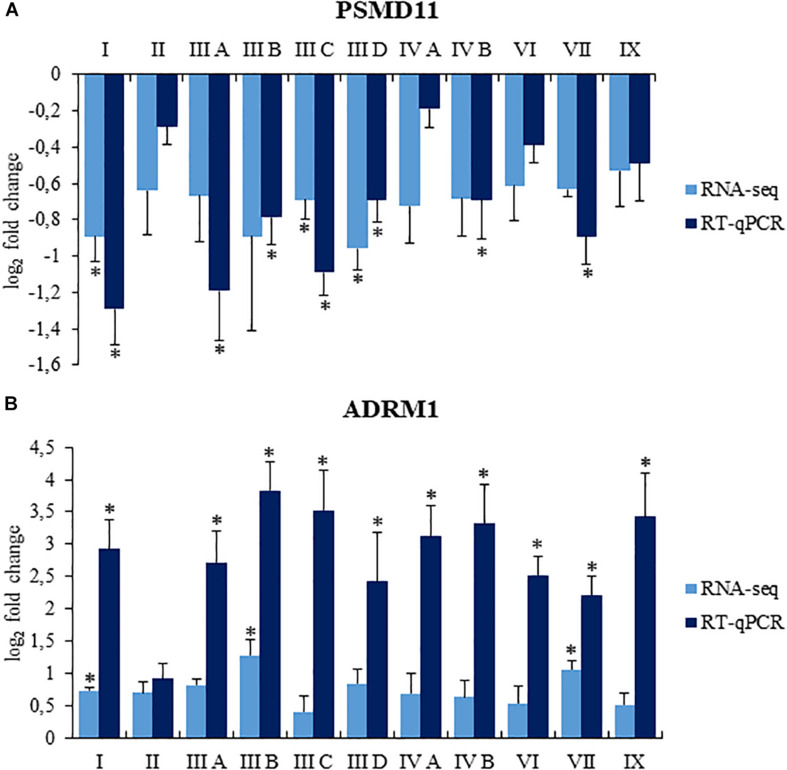
Comparison of changes in levels of selected transcripts, *PSMD11* **(A)** and *ADRM1* **(B)**, assessed by RNA-seq and RT-qPCR in MPS cells relative to control fibroblasts. Presented results are mean values from four experiments with SD shown as error bars, obtained for MPS fibroblasts relative to average values from two control cell lines (listed in section “Cell Lines”), assessed as value 0. For RT-qPCR, statistically significant differences (one-way ANOVA: Panel **(A)** F_11,24_ = 9.212, *p* = 0.001; Panel **(B)** F_11,24_ = 7.678, *p* = 0.001) relative to control cells are indicated by asterisks (statistically significant differences *p* < 0.05; Dunnett’s test).

To confirm reliability of the transcriptomic data, we have assessed levels of selected mRNAs using RT-qPCR. Abundance of transcripts derived from *ADRM1* and *PSMD11* genes was tested, and results indicated similar changes in their expression in MPS cells relative to control fibroblasts using both RNA-seq and RT-qPCR (Figure 3). Therefore, we considered that analyses of transcriptomic data are adequate when based on RNA-seq results.

As because of technical reasons only limited number of cell lines could be used in this study, for gene expression levels assessed by RT-qPCR, an additional analysis was made to confirm that the observed differences result from mean differences between the control group and MPS, and not from high variance between control cells. Detailed statistical analysis, indicating that this was the case, is presented in Supplementary Table 4.

### Proteasomal Activities in MPS Cells

**FIGURE 4:**
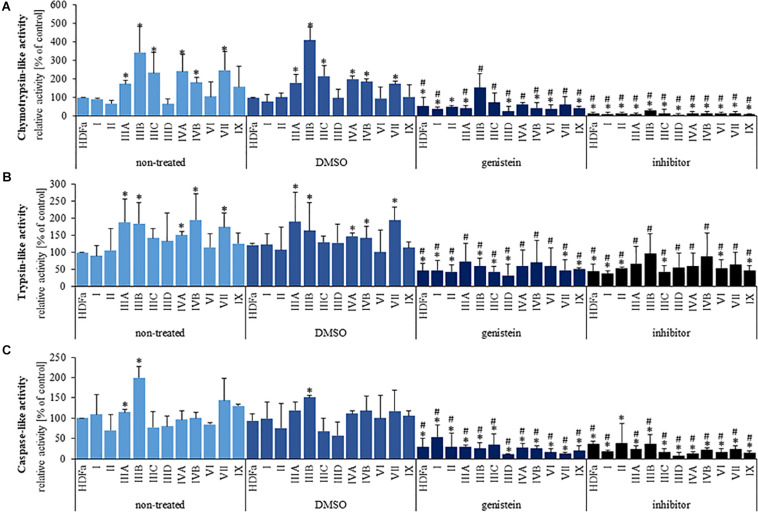
Chymotrypsin-like **(A)**, trypsin-like **(B)**, and caspase-like **(C)** 26S proteasomal activities in non-treated (control) cells and cells treated with 0.05% DMSO, 50 μM genistein, and 10 μM MG-132. Presented results are mean values from three independent experiments. Statistically significant differences relative to corresponding non-treated (or DMSO-treated in the case of genistein) cell line are indicated by hashtags (**A:** H_11,33_ = 24.089, *p* = 0.012; **B:** H_11,33_ = 11.456, *p* = 0.406; **C:** H_11,33_ = 12.899, *p* = 0.119) and those relative to control HDFa cells are indicated by asterisks (statistically significant differences *p* < 0.05; Dunnett’s test).

Since transcriptomic analyses indicated changes in expression of genes coding for proteasome-related proteins in MPS cells, we have tested if proteasomal activities are modified in these cells relative to HDFa control. Three major activities: chymotrypsin-like, trypsin-like, and caspase-like, were tested in the control cell line and in fibroblasts of all tested types/subtypes of MPS. In comparison to control cells, we have detected significantly increased chymotrypsin-like activity in MPS types IIIA, IIIC, IIIC, IVA, IVB, and VII (Figure 4A), increased trypsin-like activity in MPS types IIIA, IIIC, IVA, IVB, and VII (Figure 4B) and increased caspase-like activity in MPS types IIIA and IIIB (Figure 4C). Therefore, proteasomal activities are significantly increased in several MPS types, indeed.

**FIGURE 5:**
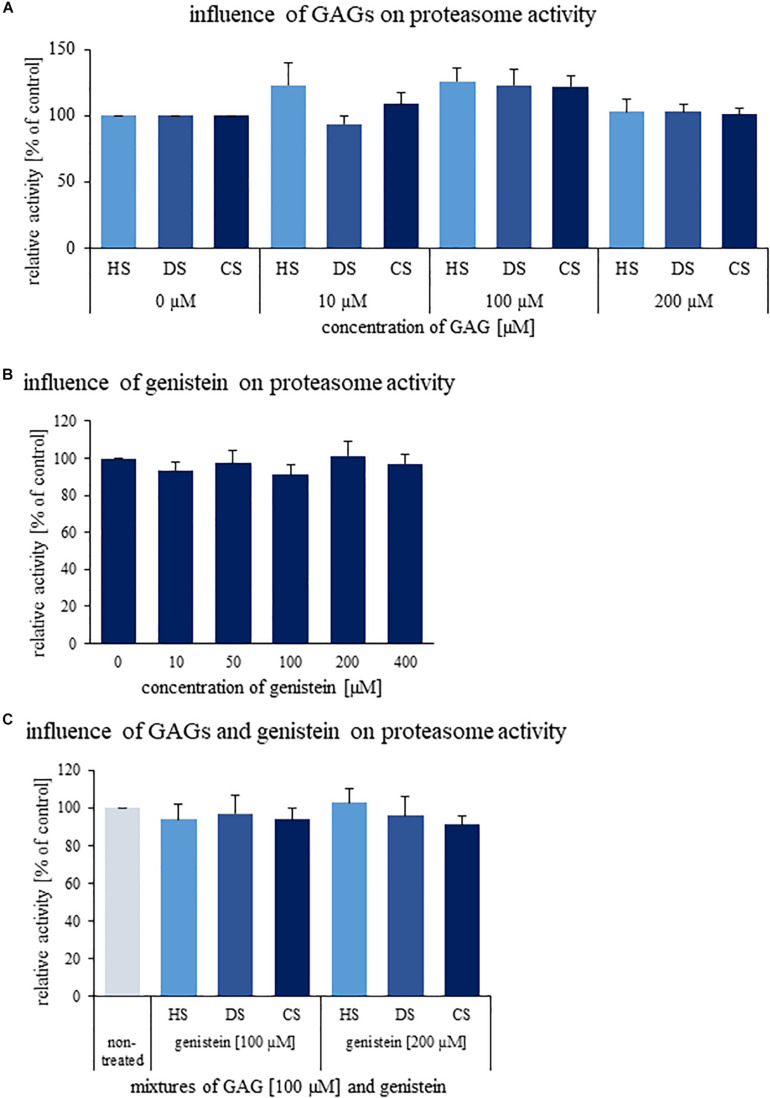
Effects of GAGs **(A)**, genistein **(B)**, and their combinations **(C)** on activity of human 26S proteasome *in vitro*. Either different kinds of GAGs (DS, HS, and CS) or genistein or both were added to indicated final concentrations and activity of human 26S proteasome was determined. Presented results are mean values from three independent experiments with error bars representing SD. Statistical analyses were performed using Kruskal–Wallis test followed by Dunn *post hoc* for Panel **(A)** (H_11,35_ = 26.655, *p* = 0.055) and Panel **(B)** (H_5,17_ = 3.001, *p* = 0.700). In turn, in the case of Panel **(C)** one-way ANOVA (F_6,19_ = 0.919, *p* = 0.510) was performed. No significant differences (*p* < 0.05) were found.

Since various GAGs are accumulated in MPS cells, we have tested if changes in proteasomal activities can be caused by direct actions of these compounds on the proteasome. However, results of *in vitro* tests indicated no significant influence of DS, HS, and CS on the activity of 26S proteasome (Figure 5A). Thus, we conclude that stimulation of the proteasomal activities in MPS cells is not a direct effect of GAG storage, and results from secondary processes occurring as metabolic consequences of the primary cause of MPS.

### Effects of Genistein on Proteasomal Activities

Genistein, a small molecule from the group of isoflavones, has been identified previously as a potential therapeutic agent for MPS, while revealing various mechanisms of biological action (see section “Introduction” for details). Moreover, a few reports indicated possible effects of genistein on proteasome (Kazi et al., 2003; Shim, 2011). Therefore, we have tested if genistein influences proteasomal activities in HDFa cells and MPS-derived fibroblasts. Chymotrypsin-like, trypsin-like, and caspase-like proteasomal activities were measured in the presence of 0.05% DMSO (solvent for genistein), 50 μM genistein, and 10 μM MG-132 – a potent inhibitor of the proteasome. We found that DMSO did not influence any proteasomal activity, while MG-132 revealed the inhibitory activity, as expected (Figure 4). However, genistein significantly reduced all tested proteasomal activities in HDFa cells and in fibroblasts derived from all MPS types, in comparison to un-treated cells (Figure 4). This indicates a potent inhibitory activity of this isoflavone against proteasomal functions in normal and MPS fibroblasts.

Some previous reports described inhibition of chymotrypsin-like activity of 20S proteasome by genistein (Kazi et al., 2003; Shim, 2011). Thus, we have tested *in vitro* effects of this isoflavone on proteasomal activity, however, experiments were performed with 26S proteasome in order to better reflect the *in vivo* conditions. As demonstrated in Figure 5B, no significant inhibition by genistein was detected for 26S proteasomal activity *in vitro*. Combinations of genistein and GAGs also did not influence 26S proteasome function (Figure 5C). Therefore, it appears that the mechanisms of inhibitory effects of this isoflavone are more complex than its direct interactions with the proteasome.

### Levels of Particular Proteasomal Subunits in MPS Cells and Effects of Genistein

Knowing that proteasomal activities are changed in MPS cells, we have asked if levels of proteasomal subunits are modulated in these cells. Therefore, using specific antibodies, we have estimated levels of particular α and β proteasomal subunits in control cells and fibroblasts derived from patients suffering from all MPS types.

**FIGURE 6:**
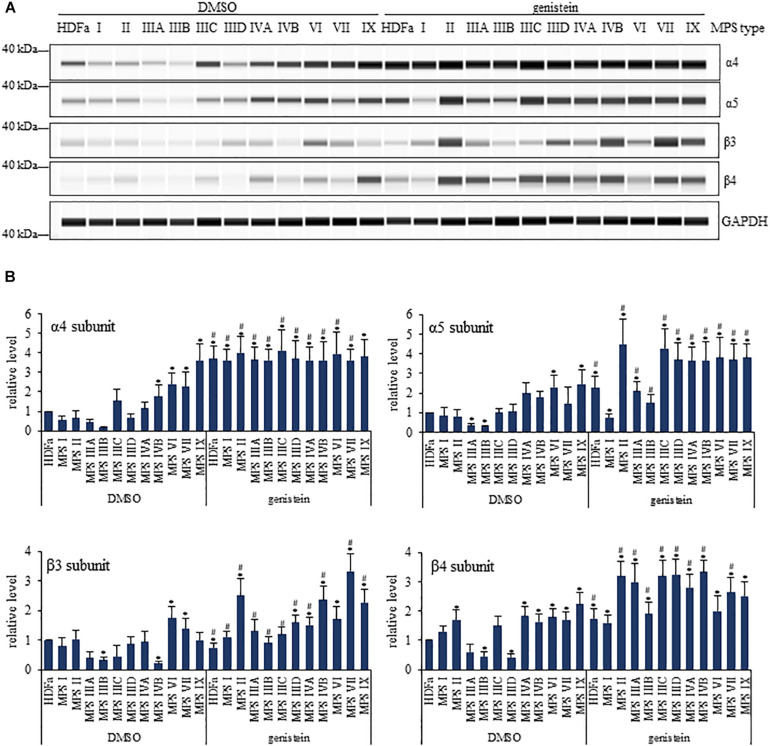
Western-blotting experiments for determination of levels of selected proteasomal subunits in control (HDFa) and MPS fibroblasts in the absence (DMSO) or presence of 50 μM genistein. The experiments were performed using the WES system. Representative blots are demonstrated in **(A)**. GAPDH was used as loading control. Quantitative analysis is presented in **(B)**. Statistically significant differences relative to control HDFa cells (values assumed to be 1) are indicated by asterisks (*p* < 0.05; Dunnett’s test), and those between genistein-treated and DMSO-treated (no genistein) cells are indicated by hashtags (for α4, one-way ANOVA F_11,24_ = 0.147, *p* = 0.049; for α5, one-way ANOVA F_11,24_ = 6.362, *p* = 0.001; for β3, one-way ANOVA F_11,24_ = 13.231, *p* = 0.001; for β4, one-way ANOVA F_11,24_ = 16.198, *p* = 0.001; followed by Tukey’s *post hoc p* < 0.05).

When assessing α subunits (representative results shown in Figure 6, and the whole set of results is demonstrated in Supplementary Figure 3), we found that α2 levels were decreased in MPS I, IIIA, IIIB, IIID and VII, α3 levels were decreased in MPS I, IIIA, IIIB, IIID and VII, α4 levels were increased in MPS VI, VII, and IX, α5 levels were decreased in MPS IIIA and IIIB while increased in MPS VI and IX, and α6 and α7 levels were decreased in MPS IIIA and IIIB. When assessing β subunits (Figure 6 and Supplementary Figure 3), we found that β1 levels were decreased in MPS I, IIIA, and IIIB, β2 levels were decreased in MPS I, IIIA, IIIB, and IIID, while increased in MPS IIIC, β3 levels were decreased in IIIB and IVB, while increased in MPS VI, β4 levels were decreased in MPS IIIB and IIID, while increased in MPS II, IVA, IVB, VI, VII, and IX, β5 levels were decreased in MPS I, IIIA, IIIB, IIID, IVB, and VI, β6 levels were decreased in MPS I, IVA, IVB, VI, VII, and IX, β7 levels were decreased in MPS I, IIIA, and IIIB, and levels of β1i and β5i were decreased in all MPS types. These results confirmed considerable changes in amounts of proteasomal subunits in various MPS types, indicating that composition of the proteasome can be significantly affected in cells of patients suffering from this disease.

In the next step, we have tested effects of genistein on levels of proteasomal subunits. We found that genistein treatment resulted in increased amounts of following α subunits in particular MPS types relative to untreated cells: α2 in MPS II, IIIA, IIIB, and IIID, α3 in MPS II, IIIA, IIIB, and IIID, α4 in all MPS types but MPS IX, α5 in all MPS types but MPS I, α6 in MPS IIIA and IIIB, and α7 in MPS IIIA and IIIB, while the only decrease was observed for α3 in MPS IX (Figure 6 and Supplementary Figure 3). Contrary to α subunits, levels of particular β subunits were either decreased or increased in genistein-treated MPS cells relative to untreated fibroblasts at the following pattern: β1 levels were decreased in MPS I, IIIA, IIIB, IIIC, IVA, and IVB, β2 levels were increased in MPS I, II, IIIA, IIIB, IIID, VII, and IX, β3 levels were increased in MPS I, II, IIIA, IIIB, IIIC, IIID, IVA, IVB, VII, and IX, β4 levels were increased in MPS II, IIIA, IIIB, IIIC, IIID, IVA, IVB, and VII, β5 levels were increased in MPS IIID, β6 levels were decreased in MPS I, II, IIIA, IIIB, IIIC, IIID, IVA, and IVB, while increased in MPS VI, VII, and IX, β7 levels were decreased in MPS IX, while increased in MPS II, IIIA, IIIB, IIIC, IIID, and IVA, β1i levels were increased in all types but MPS I, and β5i levels were decreased in MPS IX, while increased in MPS I, II, IIIA, IIIB, IIIC, IIID, IVA, IVB, and VII. Therefore, genistein considerably affected levels of various proteasomal subunits in different MPS types – in most cases, increased levels of these proteins were observed in genistein-treated MPS fibroblasts. These results indicted that inhibitory effects of genistein on proteasomal activities cannot arise directly from changes in amounts of α and β proteasomal subunits.

### Levels of Ubiquitin Conjugates in MPS Cells and Effects of Genistein

**FIGURE 7:**
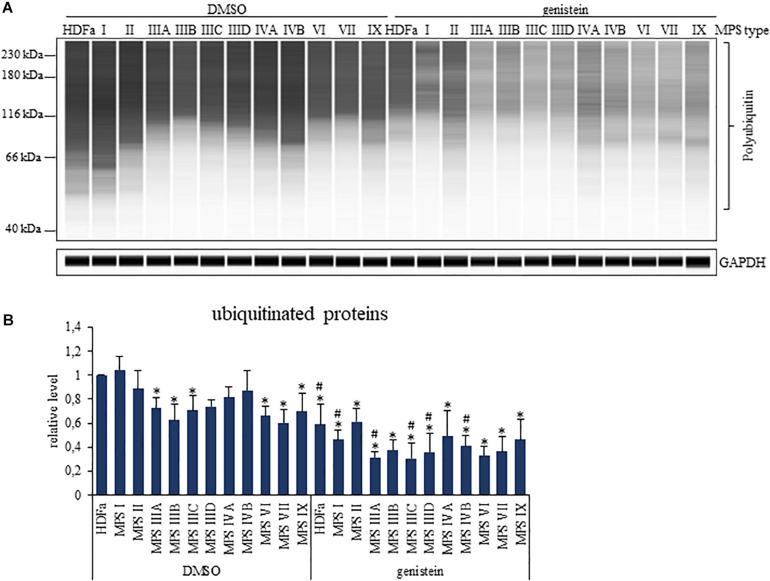
Levels of ubiquitinated proteins in control (HDFa) and MPS fibroblasts in the absence (DMSO) or presence of 50 μM genistein, assessed by western-blotting. The experiments were performed using the WES system. Panel **(A)** demonstrates representative blots in which GAPDH was used as loading control. Panel **(B)** indicates quantitative analysis of western-blotting experiments. Presented results are mean values from three independent experiments with error bars representing SD. Statistically significant differences relative to control HDFa cells (values assumed to be 1) are indicated by asterisks (*p* < 0.05; Dunnett’s test), and those between genistein-treated and DMSO-treated (no genistein) cells are indicated by hashtags (one-way ANOVA F_11,24_ = 5.134, *p* = 0.001 test followed by Tukey’s *post hoc p* < 0.05).

Since ubiquitination is a specific modification of proteins which are labeled for proteasomal degradation, and decreased levels of ubiquitin have been correlated with impaired function of the proteasome (Park et al., 2020), we have assessed levels of total ubiquitinated proteins in MPS cells. We found that levels of ubiquitin conjugates were less abundant in several MPS types relative to control fibroblasts, namely in MPS IIIA, IIIB, IIIC, VI, VII, and IX (Figure 7). Importantly, genistein caused significant lowering of amounts of ubiquitin conjugates in all tested cell lines (Figure 7). These results might suggest that decreased levels of ubiquitinated proteins may be connected to genistein-mediated reduction of proteasomal activities.

**FIGURE 8:**
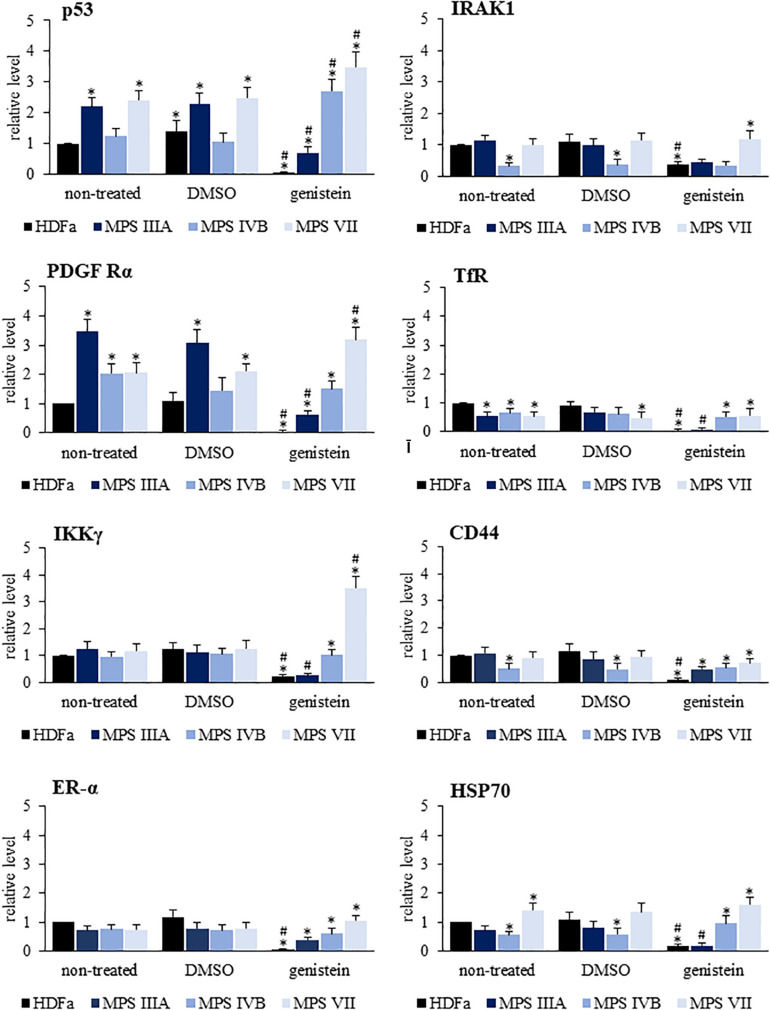
Quantification of the levels of ubiquitinated forms of eight selected proteins (out of 49 shown in Supplementary Figure 4) in HDFa cell line (control) and fibroblasts of selected MPS types, non-treated (control) or treated with either 0.05% DMSO or 50 μM genistein. The levels of ubiquitinated forms of proteins have been assessed using the Proteome Profiler Human Ubiquitin Array. Presented results are mean values from three independent experiments with error bars representing SD. Statistically significant differences relative to control HDFa cells (values assumed to be 1) are indicated by asterisks (*p* < 0.05; Dunnett’s test), and those between genistein-treated and DMSO-treated (no genistein) cells are indicated by hashtags (for p53, one-way ANOVA F_3,8_ = 32.398, *p* = 0.001; for IRAK1, one-way ANOVA F_3,8_ = 8.301, *p* = 0.008; for PDGFRα, one-way ANOVA F_3,8_ = 53.502, *p* = 0.001; for TfR, one-way ANOVA F_3,8_ = 9.158, *p* = 0.006; for IKKγ, one-way ANOVA F_3,8_ = 2.030, *p* = 0.025; for CD44, one-way ANOVA F_3,8_ = 4.536, *p* = 0.039; for ER-α, one-way ANOVA F_3,8_ = 1.369, *p* = 0.041; for HSP70, one-way ANOVA F_3,8_ = 44.735, *p* = 0.001; followed by Tukey’s *post hoc p* < 0.05).

We have also tested whether levels of ubiquitin conjugates is similar for all/most proteins or considerable differences occur for particular proteins. We used commercially available Proteome Profiler Human Ubiquitin Array to test 49 proteins, and found that there are significant differences in levels of their ubiquitinated forms in various MPS types (Figure 8 and Supplementary Figure 4). Moreover, responses to genistein were also different for different proteins, with examples of up- and down-regulation of levels of ubiquitinated forms of proteins in genistein-treated cells. Moreover, modulation of levels of ubiquitin conjugates in the case of some proteins after treatment with genistein was evident in control cells while less pronounced in selected MPS types, indicating a significant influence of metabolic changes occurring in MPS cells on the processes influencing amounts of ubiquitinated proteins and action of genistein (Figure 8). In summary, these experiments confirmed that levels of ubiquitin conjugates are considerably affected in MPS cells and genistein modifies them significantly, however, abundance of ubiquitin conjugates of particular proteins may be differentially changed in response to conditions occurring in cells of different MPS types, and can be differentially modulated by genistein.

## Discussion

Although MPS is a group of monogenic diseases (Tomatsu et al., 2018), recent studies indicated that in each MPS type there are significant changes in expression of hundreds of genes (Gaffke et al., 2020). In fact, despite GAG storage is the primary cause of the disease, secondary and tertiary metabolic changes lead to considerable disturbances in many cellular processes (summarized by Gaffke et al., 2019). Since proteasomes were not studied systematically in MPS yet, in this work we asked if composition and functions of these protein-degrading machineries are affected in all MPS types/subtypes, and if genistein, a small molecule suggested as a potential therapeutic for MPS (Wȩgrzyn, 2012) affects them.

Here, using transcriptomic analyses, we demonstrated that there is a group of proteasome-related genes which expression is significantly changed in MPS cells relative to control cell line. *HSPB11, PSMD10, PSMD11, VCP, PPP2CB, UBE2B, SPOP, UBXN8, ADRM1, UCHL1*, and *TRIM25* revealed changed expression in 3 or more MPS types, while expression of *UCHL1, FBXO38, TRIM25, CUL4A, VCP, SPOP, PSMD2, PSMD13, PSMB8, PSMB9, PSME2, HSPB1*, and *ADRM1* was changed at least twofold in at least one MPS type. These results suggested that composition and function of proteasome can be considerably affected in MPS cells. Indeed, our experimental studies demonstrated that chymotrypsin-, trypsin-, and caspase-like proteasomal activities were increased in several MPS types. These results are compatible with previous reports (describing results of studies on single MPS types) which indicated enhanced expression of genes coding for proteasomal proteins in MPS I (Khalid et al., 2016), rapid proteasomal degradation of mutant forms of iduronate-2-sulfatase in MPS II (Osaki et al., 2018; Marazza et al., 2020), rapid proteasomal degradation of mutant N-sulfoglucosamine sulfhydrolase (Muschol et al., 2011) and cysteine string protein α (CSPα) (Sambri et al., 2017), as well as elevated levels of the 19S proteasomal subunit (Beard et al., 2017) in MPS IIIA, and rapid proteasomal degradation of synaptophysin in MPS IIIB (Vitry et al., 2009). Nevertheless, results presented in this report provided global picture of proteasomal changes in all MPS types. However, these changes appear not to be direct effects of GAG storage, and in particular, putative GAG-proteasome interactions, as GAGs did not stimulate 26S proteasome activity *in vitro*. Perhaps surprisingly, western-blotting experiments indicated that despite enhanced activities of proteasomes in MPS cells, levels of several α and β subunits were either decreased or increased in different MPS types. Moreover, total level of ubiquitinated proteins was decreased in several MPS types. Although amounts of ubiquitin conjugates of individual proteins was found to be up- or down-regulated or unaffected in various MPS types, it appears that general enhancement of proteasomal activities in MPS results from complex intermolecular transactions, which perhaps involve degradation of specific misfolded or partially damaged proteins that might appear in elevated amounts in MPS cells.

Genistein is a small molecule, from the group of isoflavones, which has been considered as a potential drug for MPS (Wȩgrzyn, 2012). This compound has been demonstrated to reduce production of GAGs due to inhibition of epidermal growth factor receptor (EFGR) activity (Jakóbkiewicz-Banecka et al., 2009). Moreover, it stimulates lysosomal biogenesis by enhancing activity of transcription factor EB (Moskot et al., 2014). In fact, genistein-mediated activation of the autophagy process might contribute to its therapeutic potential in MPS (Pierzynowska et al., 2018a, 2020a). Since genistein appears to influence MPS by various mechanisms, we have asked if proteasome might also be a target for this molecule in potential treatment of this disease. In fact, all tested proteasomal activities were down-regulated by genistein in fibroblasts of all MPS types. These results are compatible with previous *in vitro* analyses which demonstrated inhibitory effects of genistein on 20S proteasome activity (Kazi et al., 2003; Shim, 2011). However, we were not able to demonstrate genistein-mediated *in vitro* 26S proteasome inhibition. These differences in *in vitro* effects of genistein most probably arise from different forms of the studied proteasome, 20S by Kazi et al. (2003) and Shim (2011), and 26S in this work.

Changes in proteasomal composition and activities are most probably secondary or tertiary effects of GAG storage in MPS cells. Since genistein reduces efficiency of GAG synthesis by inhibiting kinase activity of EFGR, and thus, impairing the signal transduction process required to stimulate expressions of genes coding for enzymes involved in GAG production (Jakóbkiewicz-Banecka et al., 2009), and this isoflavone positively regulates level and activity of transcription factor EB, a master stimulator of lysosome biogenesis (Moskot et al., 2014), one might propose that effects of genistein treatment on the proteasome are due to decrease in GAG storage, mediated by this isoflavone. However, as demonstrated in this report, genistein affects significantly proteasome functions also in control (non-MPS) fibroblast. Therefore, it is more likely that there are also genistein-mediated effects on the proteasome which are independent on reduction of GAG levels caused by this isoflavone. This concern also proteasome composition (levels of proteasomal subunits) and abundance of ubiquitin conjugates. In fact, decreased levels of ubiquitinated proteins in the presence of genistein (detected in both control and MPS cells) might result from changes in efficiencies of various processes, including ubiquitination, de-ubiquitination, and proteasomal degradation. Definitely, this report signals significant changes in proteasomal composition and functions mediated by genistein, however, more detailed molecular studies are required to elucidate precise mechanism(s) by which this isoflavone affect the proteasome.

What might be therapeutic consequences of genistein-mediated reduction of activity of the proteasome in MPS cells? Since proteasome is a protein-degrading cellular machinery, it is unlikely that it might be involved directly in GAG metabolism. However, it was reported that defective lysosomal enzymes, the products of mutated genes which are primary causes of MPS, are misfolded and therefore intensively ubiquitinated and extensively degraded by proteasomes (Vitry et al., 2009; Muschol et al., 2011; Osaki et al., 2018; Marazza et al., 2020). Therefore, any residual activity of the defective enzyme may be further reduced due to low stability of this protein. Hence, one may predict that decreased levels of ubiquitinated conjugates and resultant stabilization of the mutant enzyme should result in its increased residual activity, making degradation of GAG somewhat more efficient in cells of patients. In fact, inhibition of proteasomal degradation has been demonstrated to improve stability, translocation to lysosomes and activity of the mutant form of iduronate-2-sulfatase (an enzyme deficient in MPS II) (Osaki et al., 2018), and to normalize levels of synaptophysin which is otherwise rapidly degraded by proteasomes in MPS IIIB cells (Vitry et al., 2009). Moreover, inhibition of proteasomal activities caused normalization of levels of CSPα (a protein required for presynaptic functions) in MPS IIIA mouse neurons which were otherwise significantly reduced due to enhanced proteasomal degradation of this protein (Sambri et al., 2017). Hence, stabilization of lysosomal enzymes by impairing their proteasomal degradation has been proposed as a novel approach in treatment of MPS (Osaki et al., 2018; Marazza et al., 2020). In this light, inhibitory activity of genistein against proteasome functions may appear as another activity of this isoflavone (apart from reduction of GAG synthesis efficiency, enhancement of lysosomal biogenesis, and stimulation of autophagy) which can be beneficial for patients suffering from MPS due to modulation of biochemical pathways toward re-establishment of the metabolic balance and cellular homeostasis. One might ask if genistein-mediated reduction of proteasomal activities is safe for cells and for the whole organism. Since at the concentration used in this work, this isoflavone did not revealed cytotoxicity (Kloska et al., 2011, 2012), and it was found that genistein did not cause any significant adverse effects in pediatric patients treated for over 1 year at the dose as high as 150 mg/kg/day (Kim et al., 2013), it appears that this compound is safe at both cellular and organismal levels.

Finally, one should also remember about limitations of this study. First, only fibroblast lines were used in our experiments. This was reasonable knowing severity of the disease, low number of patients and their young age, resulting in complex restrictions related to biological material availability. On the other hand, it is worth noting that activity of the proteasome may vary between tissues, and may depend on the age and sex, even in a healthy population (Enenkel, 2014; Liepe et al., 2014). Therefore, it is crucial to indicate that our results reflect specific growth conditions of fibroblasts and might potentially differ in other cell types. Second, because of restrictions mentioned above, our control cell lines derived from adult persons while MPS patients were children (the average life span of MPS patients is below two decades), thus, the age parameter could not be normalized. Third, only one cell line of each MPS type was used, therefore, internal variations among patients suffering from the same MPS types could not be assessed. Nevertheless, the presented results indicate some common features of changes in proteasome composition and activity in most MPS types, and similar responses to genistein by various MPS fibroblasts. Thus, our results indicate specific changes in proteasomes in MPS cells, suggesting that further detailed studies should lead to understand molecular mechanisms of proteasome changes in cells of patients suffering from these diseases, and to assess efficiency of genistein to correct these disorders.

## Data Availability Statement

The datasets generated for this study can be found in the NCBI Sequence Read Archive (SRA), accession no. PRJNA562649.

## Author Contributions

KP participated in designing the experiments, performing experiments with *in vivo* proteasome activities, determination of levels of proteasomal subunits, and assessment of levels of ubiquitinated proteins, analyzed results of experiments, prepared figures and tables, and participated in writing the manuscript. LG performed experiments with RNA isolation and purification, participated in experiments with *in vivo* proteasome activities, determination of levels of proteasomal subunits, and assessment of levels of ubiquitinated proteins. EJ, JW, and EW performed *in vitro* assays probing h26S activity in the presence of GAGs and genistein. ER participated in RT-qPCR experiments and data analysis. MP performed statistical analyses. GW supervised the project, participated in designing experiments and analysis of results, and drafted the manuscript. All authors contributed to the article and approved the submitted version.

## Conflict of Interest

The authors declare that the research was conducted in the absence of any commercial or financial relationships that could be construed as a potential conflict of interest.
