# Supplementary material for: Proteasome Composition and Activity Changes in Cultured Fibroblasts Derived From Mucopolysaccharidoses Patients and Their Modulation by Genistein
Source: Front Cell Dev Biol. 2020 Oct 20;8:540726. doi: 10.3389/fcell.2020.540726 (PMC7606483; doi:10.3389/fcell.2020.540726)
Supplement: Supplementary file 1 [file Data_Sheet_1.PDF]

Figure S1

Proteasome complex (GO:0000502)

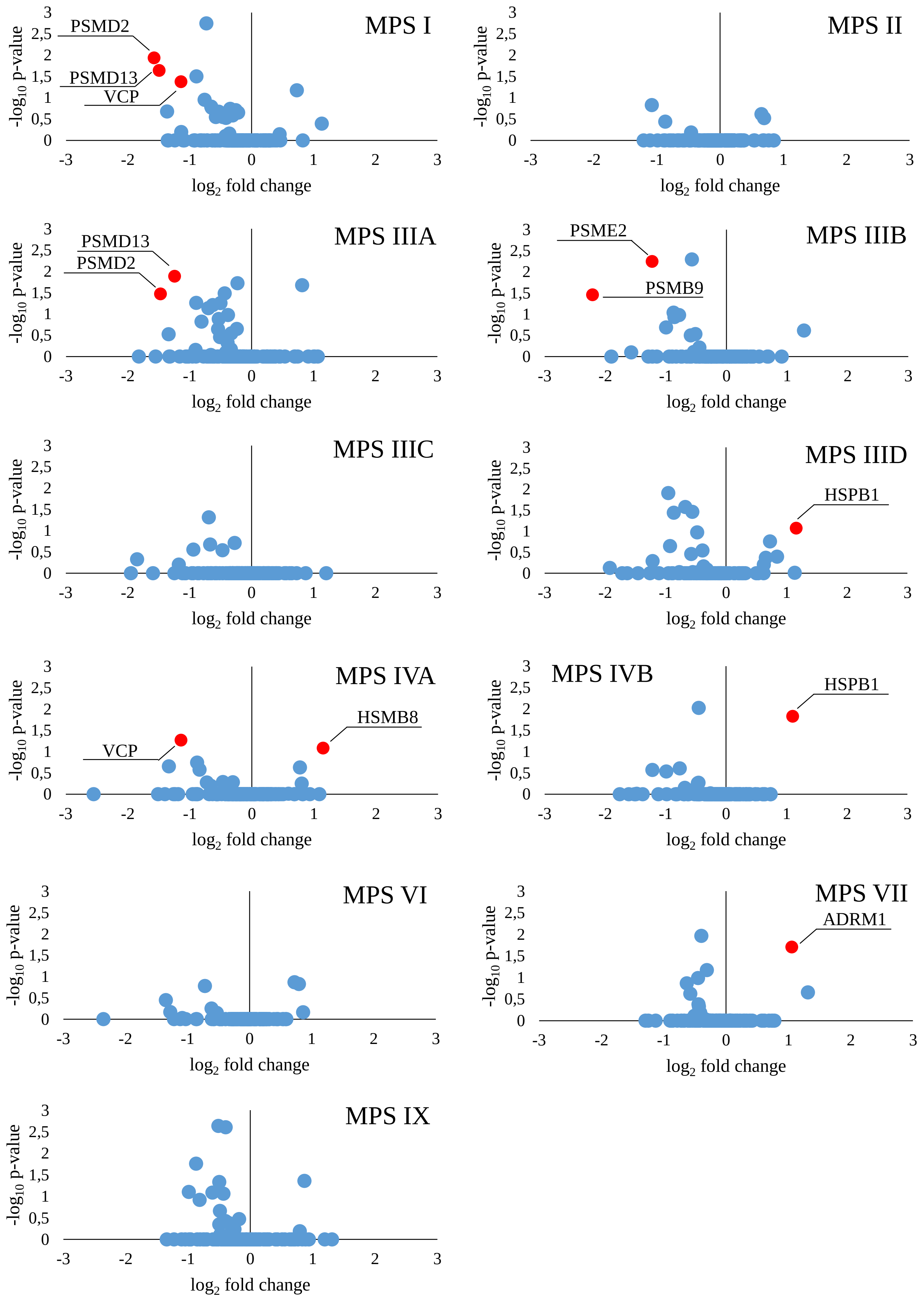

Figure S2

Proteasome-mediated ubiquitin-dependent protein catabolic process (GO:0043161)

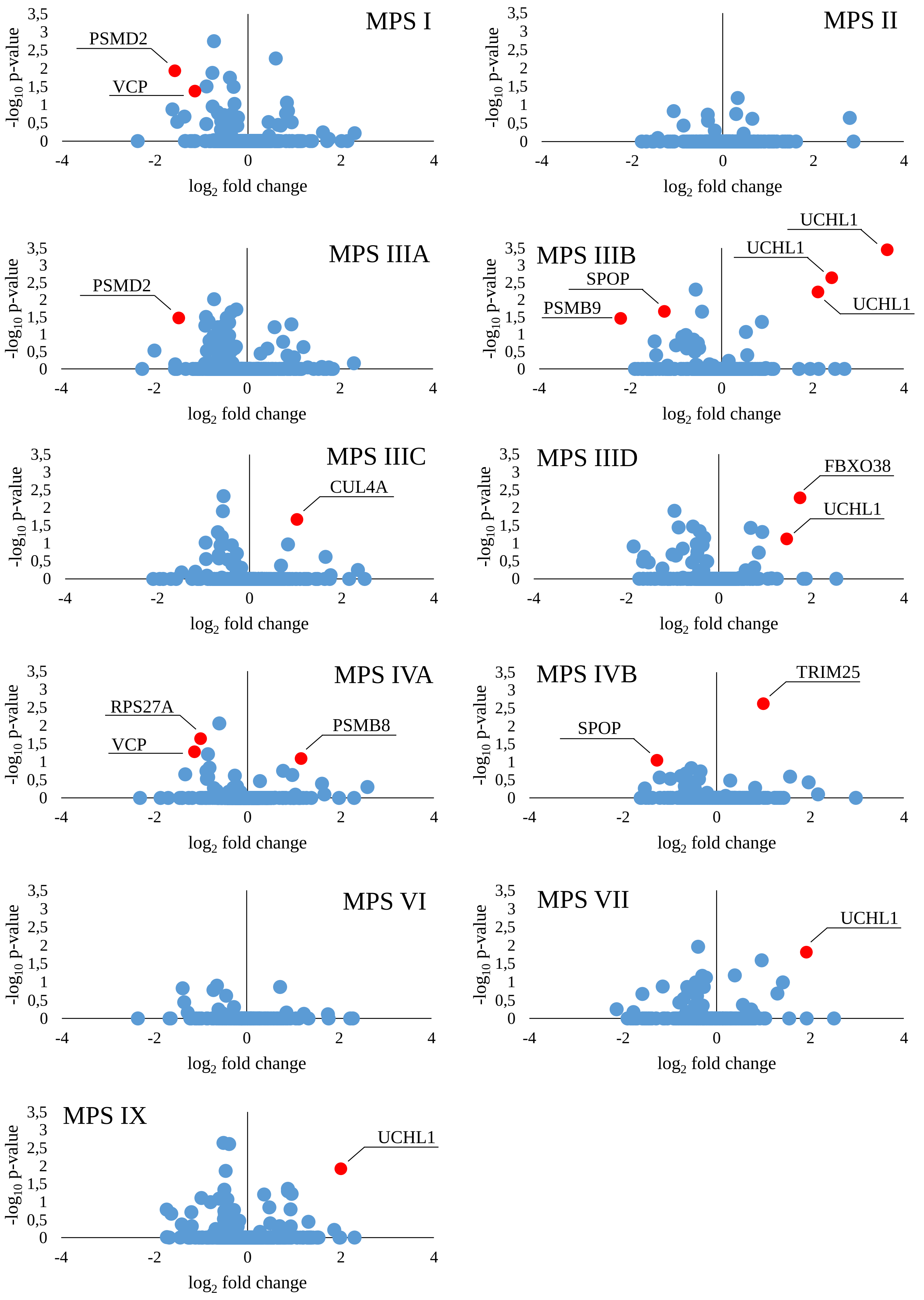

Figure S3

A

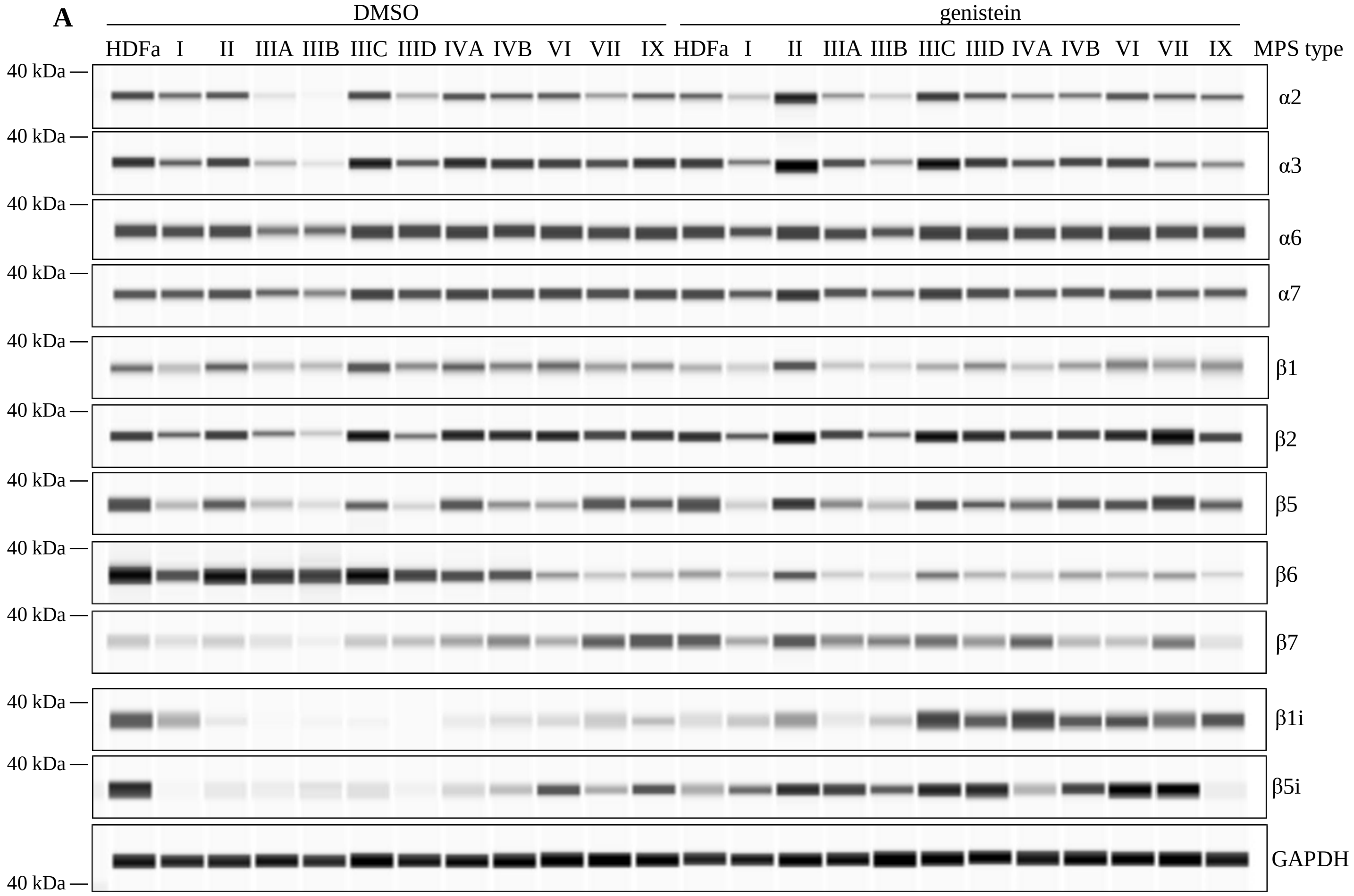

DMSO
genistein
HDFa I II IIIA IIIB IIIC IIID IVA IVB VI VII IX HDFa I II IIIA IIIB IIIC IIID IVA IVB VI VII IX
MPS type
40 kDa
α2
α3
α6
α7
β1
β2
β5
β6
β7
β1i
β5i
GAPDH

B

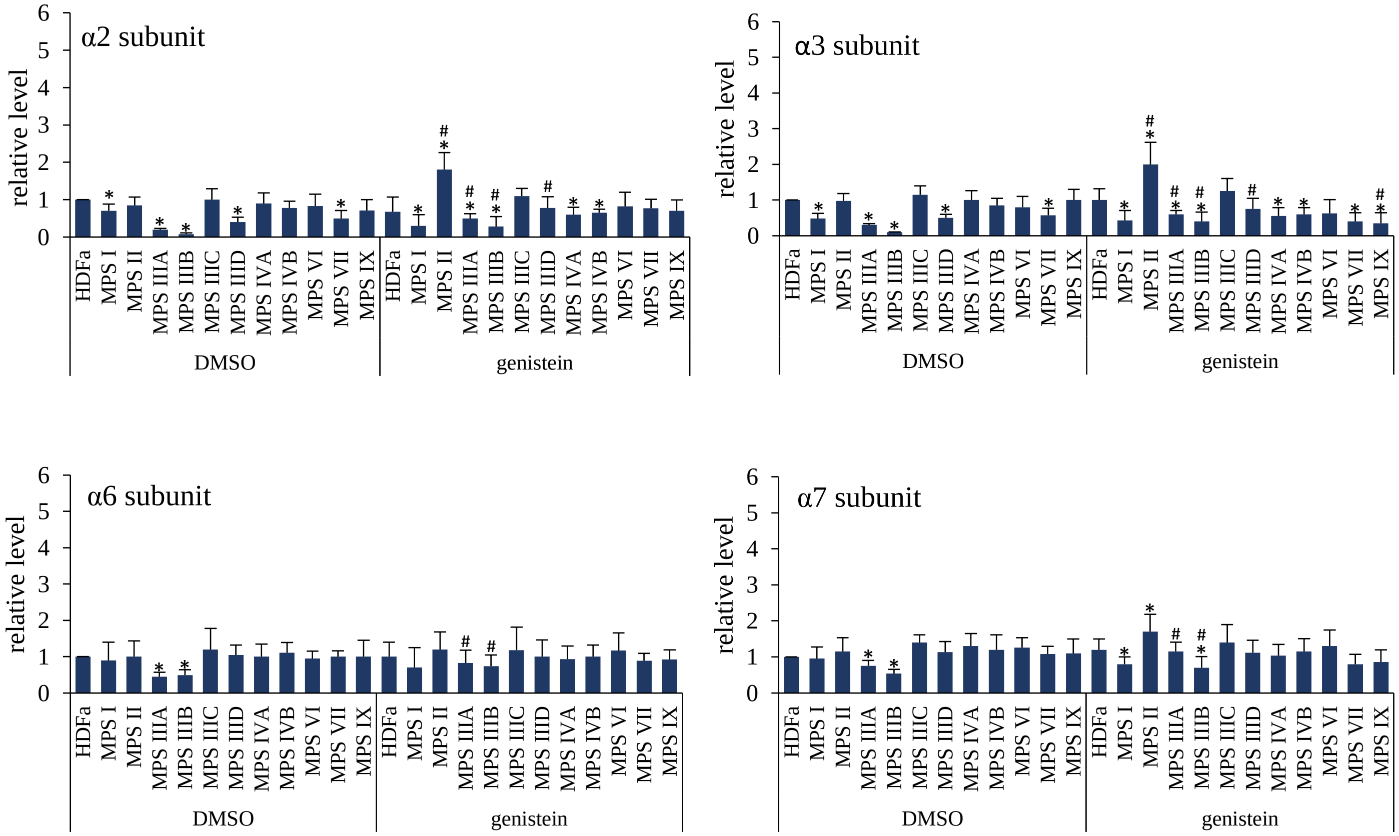

α2 subunit
α3 subunit
α6 subunit
α7 subunit
relative level
0
1
2
3
4
5
6
HDFa
MPS I
MPS II
MPS IIIA
MPS IIIB
MPS IIIC
MPS IIID
MPS IVA
MPS IVB
MPS VI
MPS VII
MPS IX
DMSO
genistein
*
#

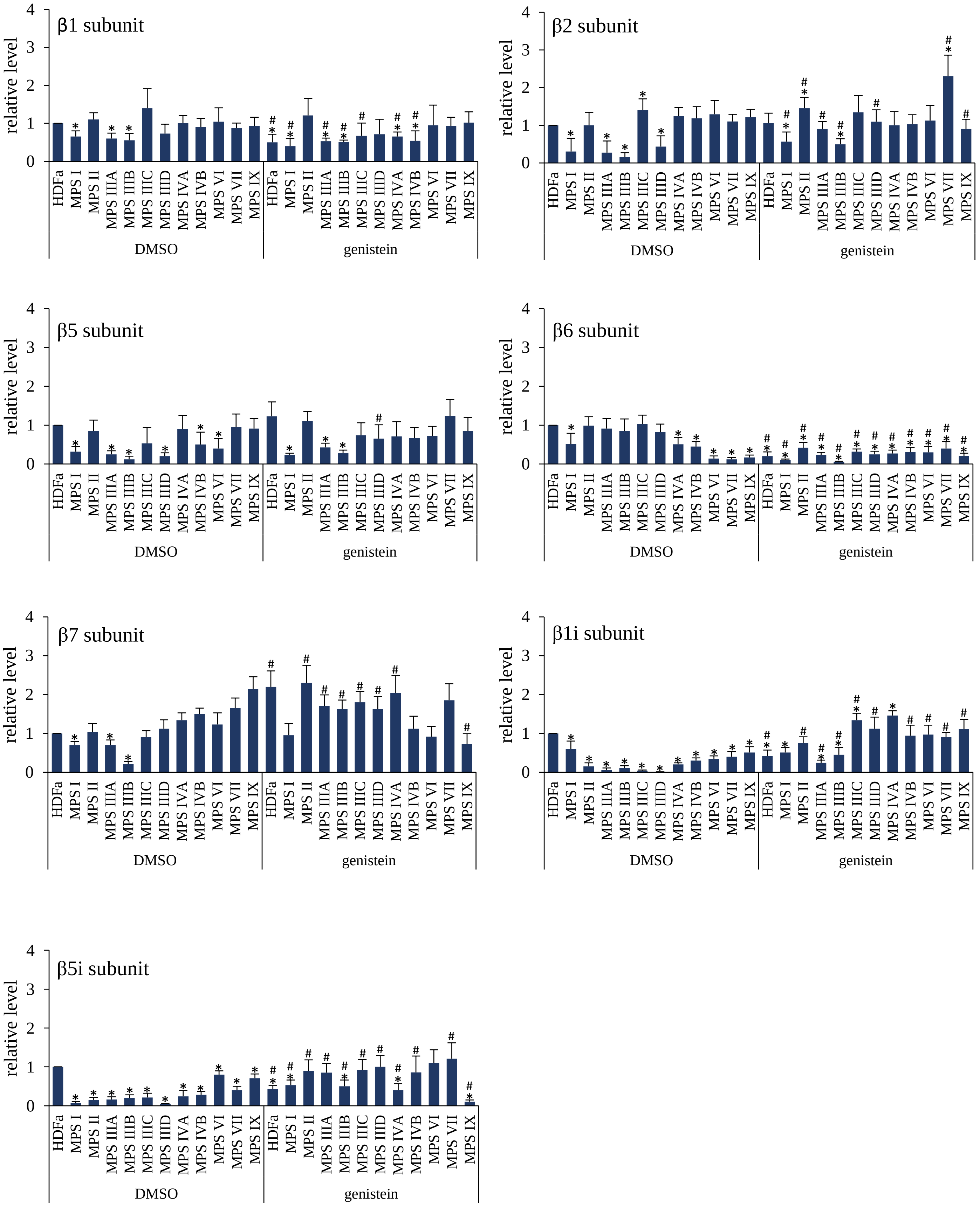
β1 subunit
β2 subunit
β5 subunit
β6 subunit
β7 subunit
β1i subunit
β5i subunit
relative level
HDFa
MPS I
MPS II
MPS IIIA
MPS IIIB
MPS IIIC
MPS IIID
MPS IVA
MPS IVB
MPS VI
MPS VII
MPS IX
DMSO
genistein

Figure S4

A

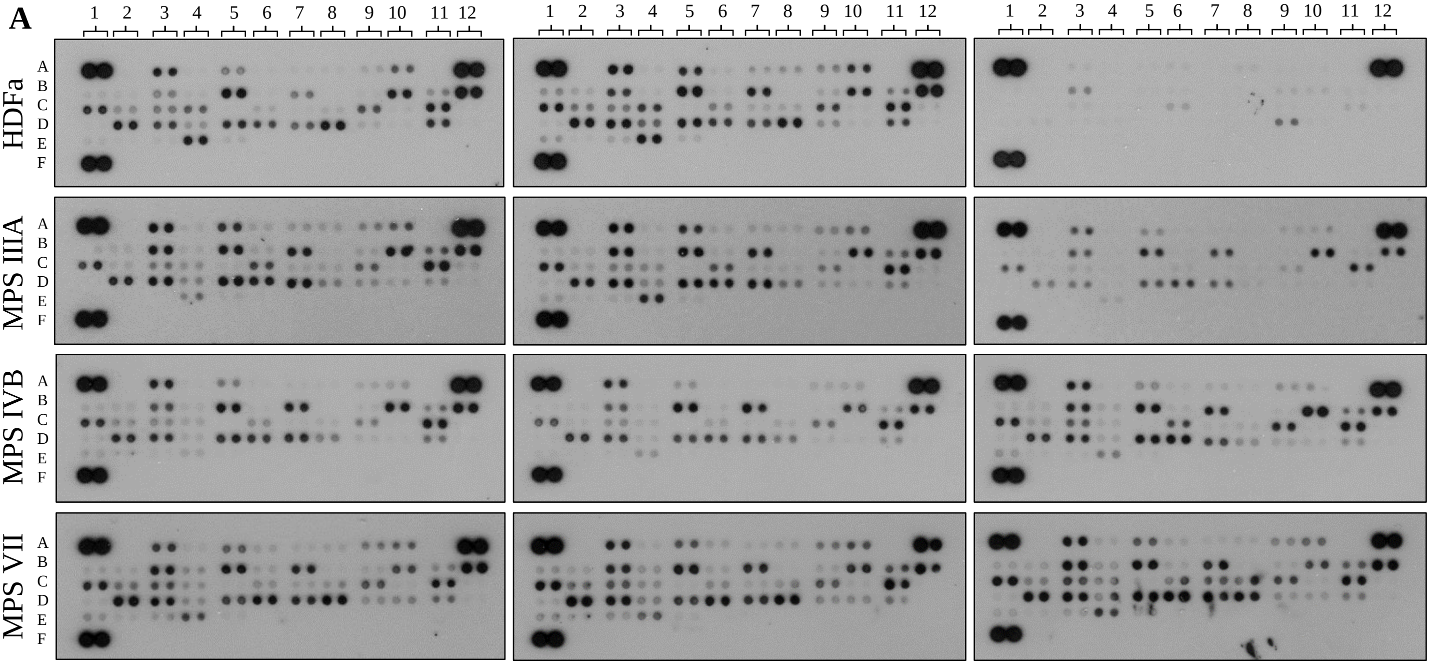

non-treated DMSO genistein

B

No.	analyte /control	entrez gene ID
A1	RS	NA
A3	A20	7128
A4	ATF4	468
A5	Bcl-2	596
A6	β-TrCP1	8945
A7	Caspase-8	841
A8	CBL	867
A9	Cyclin D1	NA
A10	CD44	595
A12	RS	NA
B1	cIAP-1	329
B2	cIAP-2	330
B3	COX-2	5743
B4	EGFR	1956
B5	ER-α	2099
B6	ErbB2	2064
B7	ErbB3	2065
B8	ErbB4	2066
B9	Fatty Acid Synthase	2194
B10	F-box protein 15	201456
B11	FBXW7	55294
B12	FGFR2α/ FGFR2β	2263
C1	HGFR	4233
C2	HIF-1α	3091
C3	HSP70	3303
C4	HSP90	3320
C5	IGF-IR	3480
C6	IκB-α	4792
C7	IκB-ε	4794
C8	IKKγ	8517
C9	Insulin R	3643
C10	IRAK1	3654
C11	IRF3	3661
C12	IRS1	3667
D1	M-CSF R	1436
D2	MSP R	4486
D3	Nrf2	4780
D4	NIK	9020
D5	p21	1026
D6	p53	7157
D7	PDGF Rα	5156
D8	PDGF Rβ	5159
D9	RIP1	8737
D10	SCF R	3815
D11	TfR	7037
D12	TNF RI	7132
E1	TRAF-2	7186
E2	TRAF-3	7187
E3	TRAF-6	7189
E4	TrkA	4914
E5	VEGF R3	2324
F1	RS	NA
F12	NC	NA

Abbreviations:
RS – reference spots
NA – not applicable
NC – negative control

Supplementary figures' legends

Figure S1. Volcano plots presenting transcripts with changed expression with log_2 fold change >1.0 or <-1.0 between control (HDFa) and MPS fibroblasts (red dots) from Proteasome complex (GO:0000502) term from QuckGO database.

Figure S2. Volcano plots presenting transcripts with changed expression with log_2 fold change >1.0 or <-1.0 between control (HDFa) and MPS fibroblasts (red dots) from Proteasome-mediated ubiquitin-dependent protein catabolic proces (GO:0043161) term from QuckGO database.

Figure S3. Western-blotting experiments for determination of levels of selected proteasomal subunits in control (HDFa) and MPS fibroblasts in the absence (DMSO) or presence of 50 µM genistein. The experiments were performed using the WES system. Representative blots are demonstrated in panel A. GAPDH was used as loading control. Quantitative analysis is presented in panel B. Statistically significant differences relative to control HDFa cells (values assumed to be 1) are indicated by asterisks ($p<0.05$; Dunnett's test), and those between genistein-treated and DMSO-treated (no genistein) cells are indicated by hashtags (one-way ANOVA test followed by Tukey's post hoc $p<0.05$).

Figure S4. Levels of 49 ubiquitinated proteins in control (HDFa) and MPS fibroblasts (selected MPS types), non-treated or treated with either 0.05% DMSO or 50 µM genistein. Levels of ubiquitinated proteins have been assessed using the Proteome Profiler Human Ubiquitin Array. Representative results are shown in panel A. The legend to identify particular dots is shown in panel B.
