# Supplementary material for: Proteasome Composition and Activity Changes in Cultured Fibroblasts Derived From Mucopolysaccharidoses Patients and Their Modulation by Genistein
Source: Front Cell Dev Biol. 2020 Oct 20;8:540726. doi: 10.3389/fcell.2020.540726 (PMC7606483; doi:10.3389/fcell.2020.540726)
Supplement: Supplementary file 2 [file Data_Sheet_2.PDF]

Table S1. Sequences of primers used in RT-qPCR.

Gene	primers (5' → 3')	
ADRM1	GGCGGGAAAGATGTCCCTG	GTCGTCCGTCTGCTGAATGT
PSMD11	GCCTCCATCGACATCCTCC	GAGCTGCTTTAGCCTTGCTG
GAPDH	GGAGCGAGATCCCTCCAAAAT	GGCTGTTGTCATACTTCTCATGG

Table S2. Statistical analyses (*p-values*) of results of transcriptomic analyses of proteasome-related transcripts which levels were changed in at least three MPS types/subtypes relative to the control cells.

Transcript	*p*-values for selected transcripts (particular MPS type vs. HDFa line)										
	I	II	III A	III B	III C	III D	IVA	IVB	VI	VII	IX
proteasome complex (GO:0000502)											
HSPB11	0.213	1.000	0.073	0.092	1.000	1.000	1.000	1.000	1.000	0.237	0.018
ADRM1	0.067	0.301	0.021	0.244	1.000	0.401	1.000	1.000	1.000	0.020	1.000
proteasome-mediated ubiquitin-dependent protein catabolic process (GO:0043161)											
PPP2CB	0.032	1.000	0.046	1.000	0.393	0.070	0.243	0.299	1.000	1.000	0.221
UBE2B	0.192	0.186	0.010	1.000	0.012	1.000	0.009	0.148	1.000	1.000	1.000
SPOP	1.000	1.000	0.733	0.022	0.097	1.000	1.000	0.090	0.881	1.000	1.000
UBXN8	0.013	1.000	0.042	1.000	0.065	1.000	0.298	1.000	1.000	0.748	0.577
UCHL1	0.087	1.000	1.000	0.006	0.814	1.000	1.000	1.000	1.000	0.103	0.051
UCHL1	1.000	1.000	0.896	0.000	1.000	0.076	1.000	1.000	1.000	0.016	0.012
TRIM25	0.005	1.000	0.360	0.043	1.000	0.037	0.343	0.002	1.000	1.000	0.060
both processes											
PSMD10	1.000	1.000	0.805	0.005	1.000	1.000	1.000	0.010	1.000	0.011	0.002
PSMD11	0.032	1.000	1.000	1.000	0.049	0.012	0.534	0.710	0.565	0.138	1.000
VCP	0.042	0.365	0.055	0.104	0.279	1.000	0.054	0.295	0.167	1.000	1.000

Table S3. Statistical analyses (*p-values*) of results of transcriptomic analyses of proteasome-related genes which transcripts occurring at levels with $log_2FC>1.0$ or <-1.0 in particular types of MPS relative to control cells (HDFa).

Transcript	*p*-values for selected transcripts (particular MPS type vs. HDFa line)										
	I	II	III A	III B	III C	III D	IVA	IVB	VI	VII	IX
proteasome complex (GO:0000502)											
PSMD13	0.023	1.000	0.013	0.798	0.471	0.748	1.000	1.000	0.937	1.000	0.121
PSME2	0.636	1.000	0.299	0.006	1.000	1.000	1.000	0.979	1.000	1.000	1.000
HSPB1	0.404	1.000	1.000	1.000	0.630	0.085	0.565	0.015	0.150	0.222	0.647
proteasome-mediated ubiquitin-dependent protein catabolic process (GO:0043161)											
SPOP	1.000	1.000	0.733	0.022	0.096	1.000	1.000	0.090	0.881	1.000	1.000
RPS27A	0.482	1.000	0.514	0.141	1.000	0.222	0.023	1.000	0.127	1.000	0.769
UCHL1	0.303	1.000	1.000	0.002	0.922	0.049	1.000	1.000	1.000	0.208	0.164
UCHL1	1.000	1.000	0.896	0.000	1.000	0.076	1.000	1.000	1.000	0.015	0.012
UCHL1	0.087	1.000	1.000	0.006	0.814	1.000	1.000	1.000	1.000	0.103	0.051
FBXO38	1.000	1.000	1.000	1.000	1.000	0.005	1.000	1.000	1.000	0.754	0.490
TRIM25	0.005	1.000	0.360	0.043	1.000	0.037	0.343	0.002	1.000	1.000	0.060
CUL4A	0.373	1.000	0.051	1.000	0.021	1.000	0.806	1.000	1.000	1.000	0.481
both processes											
VCP	0.042	0.365	0.055	0.104	0.279	1.000	0.054	0.295	0.167	1.000	1.000
PSMD2	0.012	1.000	0.034	0.206	1.000	0.226	0.267	0.271	1.000	1.000	1.000
PSMB8	1.000	1.000	1.000	1.000	1.000	0.979	0.083	1.000	0.687	1.000	0.044
PSMB9	1.000	1.000	1.000	0.035	1.000	1.000	1.000	1.000	1.000	1.000	1.000
ADRM1	0.067	0.301	0.021	0.244	1.000	0.401	1.000	1.000	1.000	0.020	1.000

Table S4. Statistical analysis of results of RT-qPCR experiments depicted in Figure 3, showing variances between two control lines (HDFa and CTRL-1) and between particular control and MPS lines.

***PSMD11* gene**

the Kolmogorov–Smirnov test: p=0.647, d=0.738	the Levene test: p=0.126, F=5.305
one-way ANOVA test: $F_{2,36}$=6.998, p=0.003<br>Tukey's post hoc test:<br>HDFa vs. CTRL-1 p=1.000<br>**HDFa vs. MPS p=0.024**<br>**CTRL-1 vs. MPS p=0.024**	

***ADRM1* gene**

the Kolmogorov–Smirnov test: p=0.210, d=1.061	the Levene test: p=0.514, F=4.780
one-way ANOVA test: $F_{2,36}$=22.842, p=0.001<br>Tukey's post hoc test:<br>HDFa vs. CTRL-1 p=1.000<br>**HDFa vs. MPS p=0.001**<br>**CTRL-1 vs. MPS p=0.001**	
